# Are major lower extremity amputations well recorded in primary care electronic health records?: Insights from primary care electronic health records in England

**DOI:** 10.1017/S1463423622000718

**Published:** 2022-11-28

**Authors:** Anna Meffen, Robert D. Sayers, Clare L. Gillies, Kamlesh Khunti, Laura J. Gray

**Affiliations:** 1 Department of Health Sciences, University of Leicester, Leicester, UK; 2 Department of Cardiovascular Sciences, University of Leicester, British Heart Foundation Cardiovascular Research Centre, Glenfield General Hospital, Leicester, UK; 3 Diabetes Research Centre, University of Leicester, Leicester, UK

**Keywords:** amputation, case ascertainment, MLEA (major lower extremity amputation), major lower limb amputation, primary care health records, surgery

## Abstract

**Aims:**

Major lower extremity amputations (MLEAs) are understood to be well recorded in secondary care in England in the Hospital Episode Statistics (HES) database. It is unclear how well MLEAs are recorded in primary care databases.

**Background:**

This study compared MLEA event case ascertainment in Clinical Practice Research Datalink (CPRD) to that in HES.

**Methods:**

MLEA events were ascertained in CPRD and in HES linkage between 1 January 2010 and 31 December 2019. The number of MLEA events and the number of patients with at least one MLEA in each database were recorded and compared. Individual events were matched between the databases using varying date-matching windows. Reasons for differences in case ascertainment were explored.

**Findings:**

In total 23 262 patients had at least one MLEA record, 8716 (37.5%) had an MLEA record in HES only, 5393 (23.2%) in CPRD only and 9153 (39.4%) in both. Out of a total of 75 221 events, 13 071 (62.4%) were recorded in HES only and 44 151 (81.3%) in CPRD only. 7874 (37.6%) of HES events were recorded in CPRD and 10 125 (18.6%) of CPRD events were recorded in HES when using the maximum date matching window of 28 days plus the time between admission and procedure. The main reasons for differences in case ascertainment included, re-recordings and miscoding in CPRD.

Compared to HES, MLEAs are poorly recorded in CPRD predominantly due to re-recordings of events and miscoding procedures. CPRD data cannot solely be relied upon to ascertain cases of MLEA; however, HES linkage to CPRD may be useful to obtain medical history of diagnoses, medication and diagnostic tests.

## Introduction

There is debate as to the number of major lower extremity amputations (MLEAs) being conducted in England, if this differs by region, and why. One reason for disagreement may be caused by the databases used to ascertain cases of MLEA (Meffen *et al*., 2021).

Surgical procedures and specifically MLEA are expected to be well recorded in the secondary care (hospital) database; Hospital Episode Statistics (HES), as hospitals receive payment for procedures performed based on events recorded in this database. Primary care databases work differently with information on secondary care-based events being manually entered into the database on the receipt of a letter informing them of an event from a hospital consultant. Methods of recording vary between primary care practices.

Whilst HES is considered the ‘gold standard’ in accuracy, its event-based nature means that it lacks the depth of patient history information that is contained in primary care data such as medications and diagnostic test results. Primary care data are also more likely to contain more accurate and up-to-date information on many chronic conditions. The increased patient history information available in primary care is important for the adjustment of patient related factors in epidemiological studies. Unlike HES, primary care data that is accessible for research purposes is available across a number of different databases covering varying proportions of the population with some overlap (NHS-Digital, 2021, 2022; CPRD, 2022; QResearch, 2022; THIN, 2022). Therefore, depending on the database used, only a subset of the population of England can be studied and whole population estimates can only be implied depending on the size and representativeness of the cohort.

A number of studies have ascertained cases of MLEA in secondary care data to estimate incidence; however, very few have ascertained cases in primary care data. This may be because it is unclear how well recorded MLEA are in primary care data and therefore also unclear if primary care data alone could be used in MLEA research studies (Vamos *et al*., 2010; Moxey *et al*., 2012; Behrendt *et al*., 2018; Gunn *et al*., 2021, Meffen *et al*., 2021). If it is possible to use primary care data when investigating MLEA epidemiology, this added depth of patient history may aid in explaining regional variations in MLEA incidence.

This aim of this study was to compare the recording of MLEA events between the secondary care database HES and the primary care database Clinical Practice Research Datalink (CPRD), using HES as the ‘gold standard’(NHS-Digital, 2022; Wolf *et al*., 2019). Ultimately, this study aims to assess whether CPRD can exclusively be used to ascertain cases of MLEA, recommending methods and highlighting limitations.

## Methods

### Study population

This study interrogated electronic health data from a subset of the population of England that were registered at a GP practice that provided data to the CPRD Aurum database and who had available HES linkage (CPRD, 2022.). Patients whose data were not defined by CPRD as meeting quality standards were excluded (CPRD, 2022). Patients aged 18 years and over who were recorded as having an MLEA due to any cause in either the linked HES and/or CPRD Aurum databases between the dates of 1 January 2010 and 31 December 2019 were included in the study.

Linkage of CPRD and HES data was performed by NHS-Digital on behalf of CPRD using NHS number, exact date of birth, sex and patient residence postcode (Padmanabhan *et al*., 2019). The linkage outcome gave each individual a patient identifier (ID) number. In this study, the patient ID was used to further link data between the two databases.

### Outcome definition

MLEA was defined as amputation of the leg above the ankle, amputations through and below the ankle were not included. Operating Procedure Codes Supplement Version 4 (OPCS-4) codes were used to ascertain cases of MLEA in HES and CPRD medcodes specific to the Aurum database were used to ascertain cases of MLEA in CPRD Aurum (*Supplement 1)* Aurum MLEA medcodes were excluded if the code term could be applied to amputations though or below the ankle. If a patient had a record for more than one MLEA in the same database, on the same date and with the same procedure code, only one of these records was included in the study; these records could represent duplicate recordings or multiple amputations (including same-day revisions and bilateral amputations). Otherwise, all other multiple event recordings per patient were included.

### Covariates

Patient demographics included age at event, sex, ethnicity, deprivation level, geographical region and whether the practice region was classified as urban or rural. Age at event was defined as the age at which the patient’s highest level amputation was performed and alongside ethnicity was taken from HES where available, and CPRD otherwise. Sex and geographical region by Strategic Health Authority of practice at which the patient was registered were taken from CPRD. Deprivation level quintiles were defined by the Index of Multiple Deprivation (IMD) 2015 based on the patient’s postcode. Urban/rural classification was produced by the Office for National Statistics using the 2011 census and was based on the postcode of the GP practice at which the patient is registered. IMD and urban/rural classification were obtained via small area level data linkage.

### Statistical analysis

The number of people with at least one event recorded in HES only, CPRD only and who had at least one recording in both databases within the study period were calculated and compared by patient demographic. The number of events in each database were calculated alongside the number and percentage of events for each level of amputation and the number of events per patient.

To ascertain whether each MLEA event recorded in CPRD was attributable to an event in HES, events were matched between the two databases using patient ID and event date. As there was likely to be some discrepancy between the date in which an event was performed in hospital and the date at which it was recorded by the patient’s GP practice, matching on exact event date recorded in either dataset may not identify all matches. Varying date windows were applied to the matching criteria to determine whether an increase in the number of matches could be achieved. The date-matching windows applied were as follows:

The observed event date in CPRD was matched to

- Exact event date in HES
- Between the HES hospital admission date and 1 week after the HES event date (7 days)
- Between the HES hospital admission date and 2 weeks after the HES event date (14 days)
- Between the HES hospital admission date and 3 weeks after the HES event date (21 days)
- Between the HfrvgES hospital admission date and 4 weeks after the HES event date (28 days)

The lower dates were chosen after initial observations of the data suggested that the observed event date in CPRD was often given as the hospital admission date. The highest date of the widest matching window was chosen to be no longer than 28 days after the event date in HES so as to attempt to avoid the possibility of falsely matching any events that occurred shortly after the previous event. As the length of time between the admission date and the event date differ by patient, the length of matching windows applied therefore varied with each patient. The number and percentage of MLEA event matches for each database were recorded, and these values were used to describe the number of events that were recorded in CPRD only, HES only and in both databases allowing for more than one event per patient. The number and percentage of matched events that agreed on MLEA level were calculated.

The event-matching process created ‘false’ and ‘duplicate’ matches, the number and percentage of these were calculated. The definitions in the context of the event date matching analysis applied are as follows:

False matches – Occurred where a single CPRD event matched to more than one HES event. This means that a patient is likely to have had more than one separate events in HES within the matching window and, only one event has been recorded for this patient in CPRD. The first occurring HES event within the matching window was used when calculating agreement of MLEA level.

Duplicate matches – Occurred when one HES event matched to multiple CPRD events. Unlike the false matches described above, these are likely to be genuine matches of recording of one event in HES to multiple re-recordings of that event in CPRD, as in CPRD, it is common for one event to have multiple re-recordings, though it is not possible to be certain. These do not need to be deducted from the total number of matches of CPRD events as they represent a match to a true event in HES, however they will need to be deducted from the total number of matches of HES events. As with the description above, when calculating the agreement in MLEA level matches, the first occurring CPRD event within the matching window was used. It was possible for a match to be both false and duplicate. We cannot be certain which of the CPRD events is the initial or most accurate recording of each separate HES event as inaccuracies in dates and MLEA level within the CPRD data are likely.

In addition to differences in patient demographics, other potential reasons for missed MLEA recordings in CPRD such as re-recordings and miscoding were explored and discussed.

### Sensitivity analysis

Preliminary investigation suggested some of the recordings in CPRD may be re-recordings of historical amputations which occurred before the index date for this study. The sensitivity analysis investigated the effect of excluding patients from the study entirely who have at least one MLEA record in CPRD only and have no record in HES within the study period but do have a record of MLEA in HES prior to the study period. Additionally, we removed events in CPRD that had a potentially historical MLEA code (as defined by * in *Supplement 1*). These were not excluded originally as preliminary investigation suggested that these codes were not always correctly used and so we decided to include these events and maximize case ascertainment. Primary results were recalculated after the removal of these patients/events.

Statistical analysis was performed in Stata Version 16.

## Results

**Figure 1:**
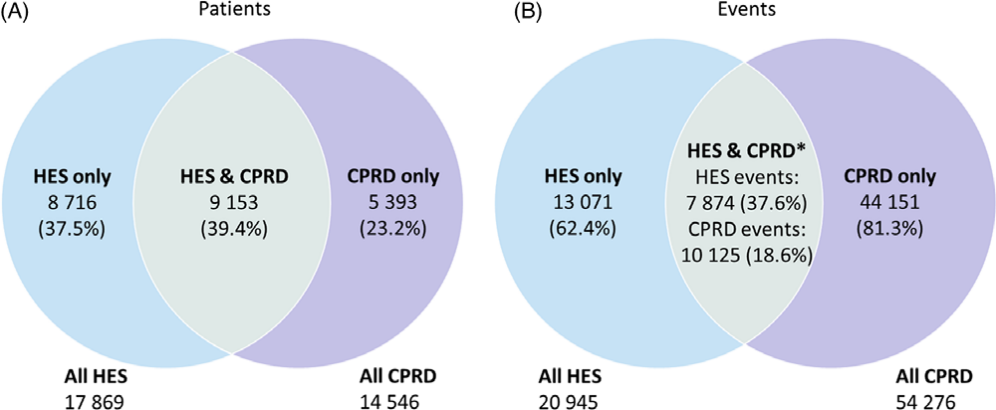
Venn diagram of (A) The number (%) of patients with at least one MLEA in HES, CPRD and patients with at least one MLEA in both HES and CPRD. (B) The number (%) of events recorded in HES, CPRD and the number of events from each database that are recorded in both. **Source**: Events were merged with the date window HES admit date to 28 days after HES event date. CPRD – Clinical Practice Research Datalink, HES – Hospital Episode Statistics, MLEA – Major lower extremity amputation. *These differ for each database as there were multiple CPRD events that relate to one HES event.

In total, 23 262 patients met the study criteria and were found to have had at least one recording of an MLEA in either CPRD or HES during the time period 1 January 2010 to 31 December 2019. 9 153 (39.4%) of these patients had a recording an MLEA in both the HES and the CPRD databases; 8 716 (37.5%) only had an MLEA recording in HES and 5 393 (23.2%) only had a recording in CPRD. Of those with any MLEA record in HES, only 51.2% (9 153 out of 17 869) also has any recording of an MLEA in CPRD within the study period. From the 23 262 patients, there were 75 221 MLEA recordings across the two databases, 20 945 in HES and over twice as many (54 276) in CPRD. 7 874 (37.6%) of events recorded in HES found a matching event record in CPRD using the maximum date matching criteria, yet, only 10 125 (18.6%) of events recorded in CPRD found a matching event record in HES (Figure 1).

### Reasons for differences in case ascertainment

When comparing the number of MLEA recordings per patient between the two databases, despite more individual patients having a recording in HES (17 869 compared to 14 546), CPRD has more event recordings (54 276 compared to 20 945). Patients with an MLEA recorded in CPRD have far more MLEA event records per patient than in HES with the maximum per patient in HES being 5 and the maximum in CPRD being 216, 2 271 (15.6%) of which have between 5 and 10 records of MLEA and 1 000 (6.8%) having more than 10. The median number of MLEA events per patient in HES is 1 compared to 2 in CPRD (Table 1).

**Table 1.** Number of MLEA records per patient in HES and CPRD

	Frequency (%)	
	HES	CPRD
Number of events		
1	15 111 (84.57)	6 095 (41.90)
2	2 475 (13.85)	3 087 (21.22)
3	250 (1.40)	1 409 (9.69)
4	31 (0.17)	1 009 (6.94)
5	2 (0.01)	675 (4.64)
>5 and <10	0 (0)	1 271 (8.80)
≥10	0 (0)	1 000 (6.81)
Median (maximum)	1 (5)	2 (216)
Number of patients	17 869	14 546

**Source:** Values presented as *n* (%) and median (maximum). Number of patients is defined as the number of patients with at least one recording of an MLEA event. CPRD – Clinical Practice Research Datalink, HES – Hospital Episode Statistics, MLEA – Major lower extremity amputation.

The cohort were predominantly male (69%) and white (86%) with a median age at procedure of 67 years. A larger majority of the cohort resided in an urban area (85%), attended a practice in the North West (21%) and had an IMD score of 3 or more (96%, more deprived) (Table 2).

**Table 2.** Patient demographic by case-ascertainment source based on the highest physical level of MLEA for each patient

	Case source			Total
	HES only	HES and CPRD	CPRD only	
Number of patients	8 719	9 153	5 393	23 262
Age at event	65 (25)	68 (21)	67 (20)	67 (23)
Sex				
Male	6 081 (69.8)	6 215 (67.9)	3 669 (68.0)	15 965 (68.6)
Female	2 635 (30.2)	2 938 (32.1)	1 724 (32.0)	7 314 (31.4)
Ethnicity				
White	7 555 (86.7)	8 223 (90.0)	4 152 (77.0)	19 930 (85.7)
Black	282 (3.2)	252 (2.8)	214 (4.0)	748 (3.2)
Asian	196 (2.3)	139 (1.5)	306 (5.7)	641 (2.8)
Other	73 (0.8)	65 (0.7)	82 (1.5)	220 (1.0)
Mixed	57 (0.7)	41 (0.5)	77 (1.4)	175 (0.8)
Index of multiple deprivation				
1 – Least deprived	1 147 (13.2)	1 341 (14.7)	688 (12.8)	3 176 (13.7)
2	1 345 (15.4)	1 563 (17.1)	782 (14.5)	3 690 (15.9)
3	1 559 (17.9)	1 759 (19.2)	905 (16.78)	4 223 (47.7)
4	1 952 (22.4)	1 971 (21.5)	1 008 (18.7)	4 931 (21.2)
5 – Most deprived	2 658 (30.5)	2 505 (27.4)	1 219 (22.6)	6 382 (27.4)
Region				
North East	386 (4.4)	491 (5.4)	177 (4.5)	1 054 (4.5)
North West	1 672 (19.2)	1 983 (21.7)	1 186 (22.0)	4 841 (20.8)
Yorkshire & Humber	343 (3.9)	403 (4.4)	408 (7.6)	1 154 (5.0)
East Midlands	219 (2.5)	213 (2.3)	98 (1.8)	530 (2.3)
West Midlands	1 366 (15.7)	1 611 (17.66)	743 (13.8)	3 720 (16.0)
East of England	337 (3.9)	387 (4.2)	223 (4.1)	947 (4.1)
South West	1 228 (14.1)	1 296 (14.2)	675 (10.1)	3 199 (14.0)
South Central	912 (10.5)	997 (10.9)	544 (10.1)	2 453 (10.6)
London	1 450 (16.6)	996 (10.9)	940 (17.4)	3 386 (14.6)
South East	798 (9.2)	776 (8.5)	387 (7.2)	1 961 (8.4)
Urban/rural classification				
Urban	7 766 (89.1)	7 924 (86.6)	4 111 (76.2)	19 801 (85.1)
Rural	950 (10.9)	1 229 (13.4)	581 (10.8)	1 760 (11.9)

**Source:** Values are presented as *n* (%) for categorical data and as median (IQR) for continuous data. Missing/unspecified values: Ethnicity – missing = 1 548 (6.7%); Deprivation – missing = 860 (3.4%); Region – missing = 17 (0.07%); Urban/rural – missing = 701 (13.0%). Deprivation quintile: 1 = least deprived; 5 = most deprived. CPRD – Clinical Practice Research Datalink, HES – Hospital Episode Statistics.

**Figure 2:**
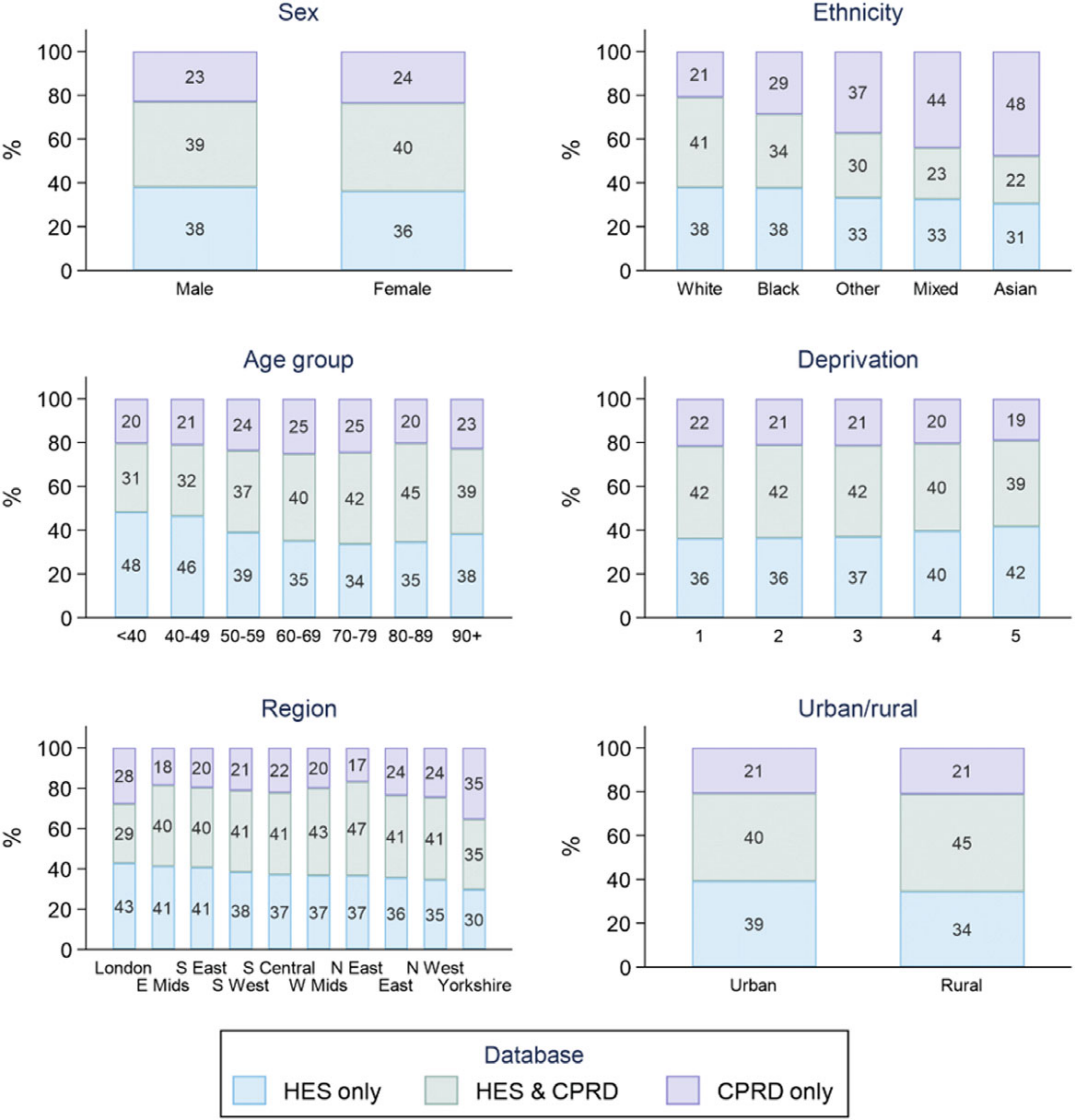
Case-ascertainment source of MLEA by patient demographic based on the highest physical level of MLEA for each patient. **Source**: Missing/unspecified values: Ethnicity – missing = 1548 (6.7%); Deprivation – missing = 860 (3.4%); Region – missing = 17 (0.07%); Urban/rural – missing = 701 (13.0%). Deprivation quintile: 1 = least deprived; 5 = most deprived. CPRD – Clinical Practice Research Datalink, HES – Hospital Episode Statistics.

Patient demographics varied minimally between those with an MLEA record in HES only, CPRD only and those with an MLEA record in both HES and CPRD. The only demographic variation of note was the difference in ethnicity proportions between those with MLEA recordings in HES only, CPRD only and both HES and CPRD. Compared to those of white ethnicity, patients of other ethnicities were more likely to have an MLEA record in CPRD only and less likely to have an MLEA record in both CPRD and HES, especially so for those of Asian ethnicity (Asian – 48% CPRD only, 22% both HES and CPRD. White – 21% CPRD only, 41% both HES and CPRD). However, the differences in ethnicity for those who have a recording in HES only are not so stark (Asian – 31% HES only, White 38% HES only) (Figure 2).

When date-matching CPRD records of MLEA events of patients that were ascertained in CPRD only to any procedure occurring on the same date in HES, it was found that in some cases other vascular lower limb procedures were performed in HES on these dates. For example, 204 people who had a record of an MLEA event in CPRD had a recording in HES for ‘re-amputation at higher level’ on the exact same date. This suggests the possibility that these procedures may have been wrongly coded in CPRD as there are separate codes available that define re-amputation that could have been used instead of the codes listed in Supplement 1 that define amputation. Other procedures recorded in HES on the exact date that could similarly be classed as being potentially miscoded in CPRD are angioplasty, bypass, revascularisation, stump revision, prosthetic related procedures and other non-specified lower limb procedures.

### Event matching

**Figure 3:**
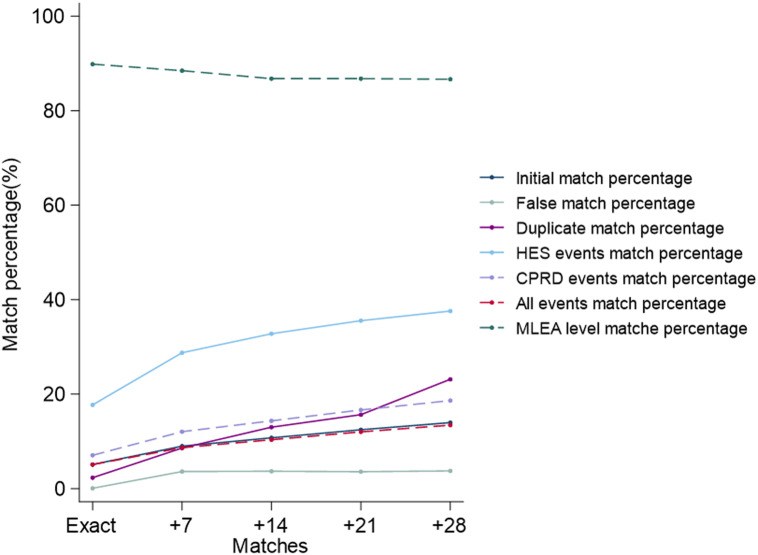
Percentage of matched events for each date matching window. **Source**: Denominators for percentages differ and are as described in Table 3. CPRD – Clinical Practice Research Datalink, HES – Hospital Episode Statistics, MLEA – Major lower extremity amputation. Total HES events = 20 945 Total CPRD events = 54 276 Total HES & CPRD events = 75 265 Date match windows are: Exact – Matched CPRD observation date on exact HES event date + 7 – Matched CPRD observation date between HES admit date and HES event date + 7 days + 14 – Matched CPRD observation date between HES admit date and HES event date + 14 days + 21 – Matched CPRD observation date between HES admit date and HES event date + 21 days + 28 – Matched CPRD observation date between HES admit date and HES event date + 28 days.

As seen in Table 3 and Figure 3, using a date-matching window as opposed to matching events on the exact HES event and CPRD observation dates increases the percentage of events matched. For example, the percentage of matched HES events increases by 61% when adding the date matching window if HES admit date to HES event date plus 7 days (*n* = 3751 (18%), plus 7 days window *n* = 6025 (29%)). The CPRD event matching percentage increases by 71% (Exact *n* = 3840 (7%), plus 7 days window *n* = 6552 (12%)). Widening this matching window has some, yet less substantial, effect. For example, the percentage of matched HES events increases by 14% when increasing the window from 7 to 14 days, 8% when increasing the window from 14 to 21 days and 6% when increasing the window from 21 to 28 days (plus 7 days *n* = 6025 (29%), plus 14 days *n* = 6867 (33%), plus 21 days *n* = 7448 (36%), plus 28 days *n* = 7874(38%)). The percentage of matched CPRD events increases by 19% when increasing the matching window from 7 to 14 days, 16% when increasing the matching window from 14 to 21 days and 12% when increasing the matching window from 21 to 28 days (plus 7 days *n* = 6552 (12%), plus 14 days *n* = 7797 (14%), plus 21 days *n* = 9050 (17%) and plus 28 days *n* = 10 125 (13%)).

**Table 3.** Results of matching MLEA events in HES to events CPRD for each match window

Matches	Exact event date match	HES admit date to event date +7 days	HES admit date to event date +14 days	HES admit date to event date +21 days	HES admit date to event date +28 days
Initial *n* (% of total HES and CPRD events combined)	3843 (5.11)	6767 (8.99)	8095 (10.76)	9387 (12.47)	10 519 (13.98)
False *n* (% of initial number of matches)	3 (0.08)	245 (3.62)	298 (3.68)	337 (3.59)	394 (3.75)
Duplicate *n* (% of initial number of matches)	89 (2.32)	581 (8.59)	1053 (13.01)	1749 (15.66)	2435(23.15)
Duplicate and false *n* (% of initial number of matches)	0 (0.00)	84 (1.24)	123 (1.52)	147 (1.57)	184 (1.75)
HES events *n* (% of HES events)	3751 (17.73)	6025 (28.77)	6867 (32.79)	7448 (35.56)	7874 (37.59)
CPRD events *n* (% of CPRD events)	3840 (7.07)	6552 (12.06)	7797 (14.35)	9050 (16.66)	10 125 (18.64)
All events *n* (% of HES and CPRD events combined)	3840 (5.10)	6522 (8.67)	7797 (10.36)	9050 (12.02)	10 125 (13.45)
MLEA level *n* (% of total HES matches)	3371 (89.86)	5331 (88.48)	5960 (86.79)	6464 (86.79)	6825 (86.67)

**Source:** CPRD – Clinical Practice Research Datalink, HES – Hospital Episode Statistics, MLEA – Major lower extremity amputation.

Total HES events = 20 945

Total CPRD events = 54 276

Total HES and CPRD events = 75 265

Whilst the percentage of false matches initially increases on implementing a matching window (Exact 0.1% to plus 7 days 3.6%) false matches do not greatly increase as the matching window increases, the number of duplicate matches does increase. Duplicate matches, however, increase on the implementation of a matching window (270% (Exact 2.3% to plus 7 days 8.6%)) and increases, though less dramatically, as the width of the date matching window increases.

In contrast, the percentage of agreement in MLEA level decreases by 1.5% on the implementation of a matching window and decreases further, at a lesser rate, as the matching window increases however agreement remains high at above 80%.

### Sensitivity analysis

1 434 (26.6%) of the patients that only had a record for an MLEA within the study period in CPRD had a record for MLEA in HES prior to the study date. This suggests that these MLEA recordings in CPRD within the study dates are likely to be re-recordings of historic amputations that actually occurred prior to the study start date. Re-recordings of historical events should be recorded using a set of codes that defined the MLEA events as historical (listed in supplement 1). However, after investigation, only 2 591 (33.3%) of the 7 785 MLEA events connected with these 1 434 patients had an MLEA recorded using a code defines as historic.

**Figure 4:**
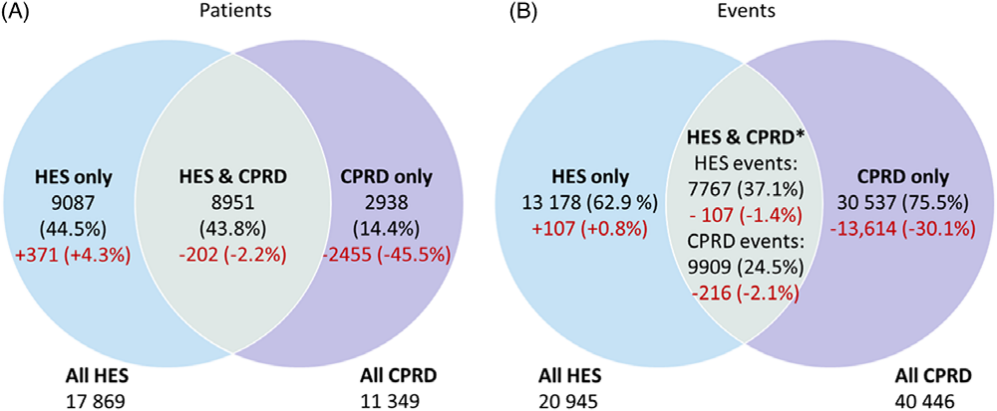
Sensitivity analysis – Venn diagram of: (A) The number (%) of patients with at least one MLEA in HES, CPRD and patients with at least one MLEA in both HES and CPRD. (B) The number (%) of events recorded in HES, CPRD and the number of events from each database that are recorded in both. Values in red show the difference (*n* (%)) from Figure 1. **Source:** Events were merged with the date window HES admit date to 28 days after HES event date. CPRD – Clinical Practice Research Datalink, HES – Hospital Episode Statistics, MLEA – Major lower extremity amputation. * These differ for each database as there were multiple CPRD events that relate to one HES event.

In total, 13 830 potentially historical MLEA events from CPRD were excluded when conducting the sensitivity analysis. 7785 CPRD events (owing to 1434 people) where the patient had no MLEA recording in HES within the study period but had an MLEA recording in HES prior to the study period were removed. Of those remaining, a further 6045 CPRD events were removed that had a potentially historical MLEA code. The values from Figure 1 have been recalculated to determine the effect of these exclusions and are shown in Figure 4 The main difference between the figure values is the percentage of patients and events recorded in CPRD only which has dropped from 23.2% to 14.4% for patients and from 81.3% to 75.5% for events. The percentage of patients recorded in both CPRD and HES rose slightly from 39.4% to 43.8% and the percentage of CPRD events that found a match in HES rose from 18.6% to 24.5%. However, the number of CPRD events that found a match in HES reduced from 10 125 to 9909, and the number of HES events that found a matching event in HES reduced from 7874 to 7767. This suggests that some true events may have been wrongly excluded as historic.

## Discussion

Compared to HES, MLEAs are not well recorded in CPRD with just over half of those recorded as having any MLEA record in HES also having a record of any MLEA in CPRD within the study period. Just over a third of the total amount of patients with at least one case of MLEA ascertained did not have any MLEA recording in CPRD within the study period meaning that using CPRD only to ascertain cases of MLEA could potentially underestimate the amount of patients affected by approximately 40%. Interestingly, 23% of all patients with at least one case of MLEA ascertained within the study period were only sourced in CPRD. When matching MLEA events between databases by exact date, only 18% of HES events and 7% of CPRD found a matching event. Increasing the matching window to match CPRD observation date to between HES admit date and HES event date plus 28 days more than doubled both the amount of HES and CPRD event matches found (to 37.6% and 18.6%, respectively). It is not clear whether these event matches are true matches as this is not possible to assess within the datasets; however, given the methods applied and the relatively high agreement in MLEA level between the two databases (86.7% using the widest date matching window), it is likely that at least the large majority of these matches are true. Despite this, the percentage of matched cases for both databases is low, particularly for events recorded in CPRD. Extending the date matching window beyond 28 days post-procedure date is unlikely to correctly match many additional events.

The predominant reason for the differences in case ascertainment is the re-recording of MLEA at subsequent GP appointments, with extremes of this reaching up to 216 records of MLEA for one patient. Although re-recordings cannot be confirmed as a certainty, over a quarter (26.6%) of patients with an MLEA ascertained only in CPRD within the study period had a HES record of an MLEA prior to the study start date suggesting that these are re-recordings of historic events. This should have been able to be confirmed by checking if the CPRD MLEA coding for these patients indicated the event was historic at the time of recording, however on interrogation, only 33.3% of the events belonging to these potentially pre-study cases were recorded as historic events. Additionally, historic MLEA event codes were not restricted to these patients and were applied when recording MLEA that were current at time of recording, as suggested by the sensitivity analysis. Relying on the application of codes that define MLEA as historic to remove potential duplicate records is therefore not possible. Had historic MLEA coding been accurate, this could also have been used to further investigate the differences in case ascertainment source by ethnicity, though as historic coding use was inaccurate, this cannot be further investigated using these data. Further investigation also discovered additional potential coding errors that may have effected case ascertainment where vascular lower limb procedures other than amputation, as recorded in HES, may have been miscoded as an amputation in CPRD. It is also possible that some GP practices use alternative methods to record MLEA other than the codes used, for example, letters from consultants informing the GP of a procedure may be written as a free-text note or scanned in and attached to a patient file meaning the procedures would be undetectable in this study. These differences in recording practice and data management between the data sources occur due to the nature and reason for data collection. HES data is collected primarily to inform payment decisions based on work done in hospitals whilst collection for data processed by CPRD is performed by GP practices to inform individual patient health care, neither of which data are specifically collected for research purposes.

Investigation suggested that demographic was not a major factor in the cause of the differences in case ascertainment. The differences seen in case ascertainment source between patients ethnicities may suggest that a small amount patients of non-white ethnicity (more so with those of Asian ethnicity) may have had an MLEA procedure abroad prior to registration at a UK GP practice though this is not possible to prove within these data. There were also small differences in case ascertainment source between GP region. These differences are likely down to differences in coding practice between GP surgeries and may be comparatively inflated in some regions where the Aurum database covers less of the population (Wolf *et al*., 2019).

Whilst this study only explored MLEA case ascertainment in one primary care database in England, this study highlights the difficulties in case ascertainment caused by recording practices of hospital-based procedures in primary care electronic health records which is applicable to many primary care services internationally.

### Limitations and justifications

This study is the first investigation of MLEA case ascertainment comparison between primary and secondary care databases. The main limitation is that it has only been conducted on a subset of the population whose anonymised data are available in the Aurum database. Despite the cohort not covering the whole population of England, the subset studied is understood to be representative of the population and covers roughly 20% of the current UK population with higher coverage in England (Wolf *et al*., 2019). Secondly, these study results are specific to the case ascertainment of MLEA and any assumptions cannot reliably be applied to case ascertainment of other surgical procedures. Thirdly, whilst all care has been taken to include all possible codes that define MLEA, it is possible that some codes may have been overlooked. In CPRD, some MLEA may be recorded using free text or uploaded documents rather than using a clinical coding system, these MLEA will not have been captured in this study. There is also the potential that some MLEA may have been miscoded using a code for minor lower extremity amputation that have not been included in this study. This study also excluded MLEA that were performed on the same date, at the same level meaning that a small amount of bilateral amputations may not have been included.

We were not able to calculate measures of agreement such as Cohen’s Kappa statistic. The unavailability of data on the non-case population (restricted by data access regulation in the case of CPRD and by the event based Nature of HES) meant that this was not possible. Although, measures of agreement statistics assume ‘rater’ (in this case data sources) independence, this is not the case in this study as recording of surgical procedures in primary care data are directly informed by secondary care data and should be recorded accordingly and so calculating these statistics in this case may not be appropriate.

### Recommendations for future MLEA research

This study recommends that future MLEA research using routinely collected electronic health records should ascertain cases of MLEA using a ‘gold standard’ secondary care dataset and use available linkage to primary care datasets to gain more detailed information on patient history if required. Researchers would need to take into consideration any limitations on population/cohort size and representativeness when applying this method. If deemed necessary, ascertaining cases of MLEA in CPRD should consider that re-recordings of events occur frequently and even after excluding events that are coded as historical, re-recordings will still occur with some true events possibly excluded. Potential ways to reduce the chances of over estimating the number of MLEA cases within a CPRD cohort would be to exclude those who have had an MLEA previous to the study period and to include only one MLEA per patient in analysis (this could be either the first occurring or highest level). However, these methods will not be suitable for all research questions, for example, it would not be possible to calculate event-based incidence of MLEA that includes multiple events per patient in CPRD alone. Researchers should consider the possibility and effects of miscoding (as minor amputations and/or other vascular procedures) and non-coded database input methods.

## Data Availability

Data used in this study are accessible only on approval of application via CPRD’s Research Data Governance (RDG) Process and cannot be publicly shared. Code scripts used in analysis can be made available on request by contacting the corresponding author.

## Supplementary material

For supplementary material accompanying this paper visit http://doi.org/10.1017/S1463423622000718. click here to view supplementary material

## Summary

To summarize, when compared to HES, MLEA are poorly recorded in CPRD predominantly due to re-recordings of events, miscoding procedures and recording method. CPRD data cannot solely be relied upon to ascertain cases of MLEA, though linkage of secondary care data to CPRD for medical history related to diagnoses and diagnostic testing would undoubtedly be useful.

## Preregistration

No preregistration exists for the reported studies reported in this article.

## References

[ref1] Behrendt CA , Sigvant B , Szeberin Z , Beiles B , Eldrup N , Thomson IA , Venermo M , Altreuther M , Menyhei G , Nordanstig J , Clarke M , Riess HC , Bjorck M and Debus ES (2018) International variations in amputation practice: A VASCUNET report. European Journal of Vascular and Endovascular Surgery 56, 391–399. 29859821 10.1016/j.ejvs.2018.04.017

[ref2] CPRD (2022) *CPRD Aurum March 2021 dataset* [Online]. Available: https://cprd.com/cprd-aurum-march-2021

[ref3] CPRD (2022) *Clinical practice research datalink* [Online]. Available: https://cprd.com/

[ref4] Gunn LH , Vamos EP , Majeed A , Normahani P , Jaffer U , Molina G , Valabhji J and Mckay AJ (2021) Associations between attainment of incentivized primary care indicators and incident lower limb amputation among those with type 2 diabetes: a population-based historical cohort study. BMJ Open Diabetes Research & Care, 9. 10.1136/bmjdrc-2020-002069 PMC8076942 33903115

[ref5] Meffen A , Houghton JSM , Nickinson ATO , Pepper CJ , Sayers RD and Gray LJ (2021) Understanding variations in reported Epidemiology of major lower extremity amputation in the UK: a systematic review. BMJ Open 11, e053599. 10.1136/bmjopen-2021-053599 PMC8496376 34615685

[ref6] Moxey PW , Hofman D , Hinchliffe RJ , Poloniecki J , Loftus IM , Thompson MM and Holt PJ (2012) Delay influences outcome after lower limb major amputation. European Journal of Vascular and Endovascular Surgery: The Official Journal of the European Society for Vascular Surgery 44, 485–490. 22967904 10.1016/j.ejvs.2012.08.003

[ref7] NHS-DIGITAL (2021) *General Practice Extraction Service* [Online]. Available: https://digital.nhs.uk/services/general-practice-extraction-service#how-gp-collections-work

[ref8] NHS-DIGITAL (2022) *Hospital Episode Statistics (HES).* [Online]. Available: https://digital.nhs.uk/data-and-information/data-tools-and-services/data-services/hospital-episode-statistics

[ref9] OPEN-SAFELY (2022) *Secure analytics platform for NHS electronic health records* [Online]. Available: https://www.opensafely.org/

[ref10] Padmanabhan S , Carty L , Cameron E , Ghosh RE , Williams R and Strongman H (2019) Approach to record linkage of primary care data from Clinical practice Research Datalink to other health-related patient data: overview and implications. European Journal of Epidemiology 34, 91–99. 30219957 10.1007/s10654-018-0442-4 PMC6325980

[ref11] QRESEARCH (2022) *Generating New Knowledge To Improve Patient Ccare* [Online]. Available: https://www.qresearch.org/

[ref12] THIN (2022) *THIN* – *The Health Improvement Network* [Online]. Available: https://www.the-health-improvement-network.com/?hsLang=en

[ref13] Vamos EP , Bottle A , Edmonds ME , Valabhji J , Majeed A and Millett C (2010). Changes in the incidence of lower extremity amputations in individuals with and without diabetes in England between 2004 and 2008. Diabetes Care 33, 2592–2597. 20833865 10.2337/dc10-0989 PMC2992196

[ref14] Wolf A , Dedman D , Campbell J , Booth H , Lunn D , Chapman J and Myles P (2019) Data resource profile: clinical Practice Research Datalink (CPRD) Aurum. International Journal of Epidemiology 48, 1740–1740g. 30859197 10.1093/ije/dyz034 PMC6929522

