# Supplementary material for: Are major lower extremity amputations well recorded in primary care electronic health records?: Insights from primary care electronic health records in England
Source: Prim Health Care Res Dev. 2022 Nov 28;23:e77. doi: 10.1017/S1463423622000718 (PMC9706375; doi:10.1017/S1463423622000718)
Supplement: Supplementary file 1 [file S1463423622000718sup001.docx]

Supplement 1: Codelists

Table 1: Aurum medical codes used to ascertain MLEA cases in CPRD Aurum.

| Medcode (Aurum) | V2 Term |
| --- | --- |
| 394601014 | Hindquarter amputation |
| 668831000006118 | Ferre hindquarter amputation |
| 805681000006117 | Gordon-Taylor hindquart amput |
| 758211000006112 | Jaboulay hindquart amputation |
| 754281000006113 | King hindquarter amputation |
| 132291000006113 | Sorrondo hindquarter amputat |
| 125921000006113 | Steelquist hindquarter amputat |
| 107671000006115 | Taylor hindquarter amputation |
| 64803017 | Disarticulation of hip |
| 523771000006116 | Boyd disarticulation of hip |
| 761121000006112 | Fitzmaurice - Kelly disarticulation of hip |
| 480191000006114 | Amputation above knee |
| 754301000006112 | Kirk thigh amputation |
| 480361000006115 | Thigh amputation |
| 451400013 | O/E-Amputated right above knee (on examination)* |
| 451401012 | O/E-Amputated left above knee (on examination)* |
| 480501000006114 | Amputation through knee |
| 504001000006119 | Batch disarticulation of knee |
| 533001000006110 | Callander disarticulation knee |
| 1785737016 | Disarticulation of knee |
| 807251000006116 | Gritti-Stokes disartic knee |
| 754311000006110 | Kirk disarticulation of knee |
| 715391000006114 | Mazet disarticulation of knee |
| 714531000006117 | McFaddin disarticulation knee |
| 136751000006110 | Slocum disarticulation of knee |
| 135581000006117 | Spittler disarticulation knee |
| 480201000006112 | Amputation below knee |
| 523751000006114 | Boyd amputation through tibia |
| 527701000006116 | Burgess below knee amputation |
| 807811000006118 | Guyon amputation lower leg |
| 451402017 | O/E-Amputated right below knee (on examination)* |
| 451403010 | O/E-Amputated left below knee (on examination)* |
| 1785726017 | Amputation of leg |
| 278463015 | Amputation of leg OS (other specified) |
| 278464014 | Amputation of leg NOS (not otherwise specified) |
| 251861017 | H/O: lower limb amputation (history of)* |
| 451398014 | O/E - Amputated right leg (on examination)* |
| 451399018 | O/E - Amputated left leg (on examination)* |

*Potentially historic events

Table 2: OPCS-4 procedure codes used to ascertain cases of MLEA in HES linkage

| OPCS-4 code | Definition |
| --- | --- |
| X091 | Hindquarter amputation |
| X092 | Disarticulation of hip |
| X093 | Amputation of leg above knee |
| X094 | Amputation of leg through knee |
| X095 | Amputation of leg below knee |
| X098 | Other specified |
